# A Phase I, Randomized, Single-Dose Study to Evaluate the Biosimilarity of QL1206 to Denosumab Among Chinese Healthy Subjects

**DOI:** 10.3389/fphar.2020.01329

**Published:** 2020-10-08

**Authors:** Hong Zhang, Min Wu, Xiaoxue Zhu, Cuiyun Li, Xiaojiao Li, Jixuan Sun, Chengjiao Liu, Quan Liu, Wei Wei, Junqi Niu, Yanhua Ding

**Affiliations:** ^1^Phase I Clinical Research Center, The First Hospital of Jilin University, Jilin, China; ^2^Institute of Translational Medicine, The First Hospital of Jilin University, Changchun, China; ^3^Institute of Virology and AIDS Research, The First Hospital of Jilin University, Changchun, China; ^4^Department of Hepatology, The First Hospital of Jilin University, Changchun, China

**Keywords:** ****denosumab, biosimilar, immunogenicity, pharmacokinetics, pharmacodynamics, inter-subject variability

## Abstract

**Objective:**

This study was conducted to explore the tolerance, variability, pharmacokinetics (PK), and pharmacodynamics (PD) of denosumab biosimilar (QL1206) in healthy Chinese subjects.

**Methods:**

This is a randomized, double-blind, two-arm, parallel study performed to examine the bioequivalence of denosumab biosimilar, QL1206, with that of Xgeva^®^ (Denosumab) as a reference drug. A single dose of 120 mg/kg of the denosumab biosimilar or Xgeva^®^ was administered to the subjects, who were followed up for 134 days.

**Results:**

Similar PK properties as those of Xgeva^®^ were exhibited by QL1206. When compared to QL1206 with Xgeva^®^, the 90% confidence intervals of the ratios for C_max_, AUC_0-t_, and AUC_0-∞_ were observed to be within 80–125%. The inter-subject variability (inter-CV) ranged from 29% to 39.5%. Six and three subjects in the QL1206 and Xgeva^®^ groups were found to be positive for the ADA and negative for the NAb, respectively. The CTX1 concentration-time profiles appeared similar (about 80% decrease from 48 hours to134 days) between the QL1206 and Xgeva^®^ groups. Adverse events (AEs) were observed in 92.6% and 93.4% of subjects in the QL1206 and Xgeva^®^ groups, respectively. Reduction in blood calcium level was found to be the most common AE recorded, with an incidence of 72.8% versus 72.4% in the QL1206 and Xgeva^®^ groups, respectively.

**Conclusion:**

Similar PK and PD characteristics were exhibited by QL1206 as compared to those of Xgeva^®^. The inter-CV was slightly large. The safety profiles of denosumab biosimilars and Xgeva^®^ were found to be similar.

## Introduction

A biologic drug is a large, complex molecule produced from living cells. Biological products that are extremely similar to another natural reference product in safety and efficacy are termed biosimilars (EMA, 2014). The innate complexity of biologics renders them difficult to be manufactured, and ineffective end products may be produced with minor variations in the manufacturing process, otherwise termed product divergence [WHO, 2010; FDA(US), 2015].

Denosumab binds to the receptor activator of nuclear factor kappa-B ligand (RANKL), a transmembrane or soluble protein that plays an indispensable role in the formation, function, and survival of osteoclasts, which are responsible for bone resorption. The binding could likely reduce bone resorption and increase bone mass, thus strengthening both cortical and trabecular bones. Denosumab (Xgeva^®^) is a specific antibody for RANKL (AmgenXgeva, 2020). Denosumab has been permitted to be used in the treatment of several bone disorders among postmenopausal women with osteoporosis at a high risk for fracture, osteoporosis, cancer-related bone disease, and so on in the United States, Europe, and Japan, excepting in China.

China and other global regions are actively developing denosumab biosimilars. QL1206 possesses an identical primary structure to that of the denosumab reference product (Xgeva^®^), and the posttranslational modifications, biochemical properties, and biological functions are similar too. The resemblance of denosumab biosimilar (QL1206) to the denosumab reference product has also been evidenced in *in vivo* studies that supported the clinical development of these denosumab biosimilars, such as preclinical pharmacokinetic (PK), pharmacodynamics (PD), and pharmacological toxicological studies that compared QL1206 and Xgeva^®^ (data not provided).

PK studies in humans are essentially conducted to validate the bioequivalence between a biosimilar and a reference product [FDA(CHINA), 2015]. The bioequivalence between denosumab biosimilar (QL1206) and Xgeva^®^ as a reference product in a single-dose PK study in healthy Chinese subjects has been examined in this study. The single-dose study design must aid in detecting intrinsic differences in the PK profiles between the denosumab biosimilar (QL1206) and the denosumab reference product. A healthy study population avoids confounding factors such as the variability associated with disease conditions, comorbidities, and concomitant therapies. A therapeutic dosage of 120 mg of the reference drug is recommended for the prevention of bone-related diseases in patients with multiple myeloma and solid tumors (AmgenXgeva, 2020). The dosage of 120 mg/kg was used in this study based on earlier clinical trial plans of the sponsor.

Here the PK profile of denosumab 120 mg, also known as Xgeva^®^ (Amgen Manufacturing Limited, Thousand Oaks, California), was compared with that of QL1206 (Socinski et al., 2015). The denosumab biosimilars were also assessed for their tolerability, safety, and immunogenicity, as well as the PD profile.

## Methods

### Study Design and Subjects

This study was carried out in the Phase I Clinical Research Center of the First Hospital of Jilin University between 2018/04/03 and 2019/05/22 (Clinical Trial Registry: NCT03651947; Chinese Clinical Trial Registry, Registration No. CTR20181448). The Institutional Review Board of the First Hospital of Jilin University reviewed and approved the final protocol, any amendments, and informed consent documentation. The study agreed with guidelines of the Declaration of Helsinki, International Conference on Harmonization Good Clinical Practice Guidelines, and local regulatory requirements. Written informed consent was obtained from all subjects prior to including them in the study.

The inclusion criteria for this phase I, double-blind, randomized, parallel-group, single-dose, two-arm study are as follows: Healthy females or males aged 18–50 years, with a body mass index of 18.0–28.0 kg/m^2^ and a total body weight of more than 50 to 85 kg, were registered in the study. Subjects had a clear medical history with no clinically relevant abnormal results of laboratory tests, physical examination, or electrocardiography (ECG).

The exclusion criteria involved expelling from the study subjects with earlier or ongoing osteomyelitis or ONJ (jaw necrosis), odontopathy, or maxillary disease in an active stage needing oral surgery (**Supplementary Materials**).

The screening visit was scheduled 14 days before the date of the dosing. Post-screening, the subjects were admitted to the Clinical Research Unit a day prior to the administration of the denosumab biosimilars. The subjects fasted for at least 8 hours (h) before dosing and were randomized into two groups: the test drug (T) and reference drug (R) groups in a 1:1 ratio. SAS 9.4 statistical analysis software (Cary, NC, USA) was used to stratify subjects according to sex and weight (stratified as ≥66 kg and <66 kg). A random table was generated for each stratification using random block, and subjects were assigned to the test or reference group in a 1:1 ratio to minimize interindividual differences in each group. A single dose of the denosumab biosimilar (120 mg QL1206, Qilu pharmaceutical co. LTD, Batch number: 201702001 KJH) was administered to the T group subjects and an equivalent dose of denosumab (Xgeva^®^, Amgen Pharmaceutical Co. Ltd., Batch number: 1080217) through a subcutaneous injection at 3–5 cm within the periumbilical region. Subjects were discharged 3 days post-dosing and followed up on days 4 to 134 as an outpatient for additional analyses in the clinical research unit. Blood samples for the primary PK and PD analyses were collected prior to the commencement of treatment and through the final follow-up. At each time point, 3.5, 4, and 3.5 ml venous blood were collected for serum denosumab concentration, immunogenicity evaluation, and pharmacodynamics evaluation (CTX1), respectively.

### PK Evaluations

Blood samples for PK evaluation were collected at 0.5 h prior to dosing (pre-dose) and to D134 days post-dosing (**Supplementary Materials**). The serum concentrations of denosumab biosimilar (QL1206) and denosumab (Xgeva^®^) were analyzed with the help of an enzyme-linked immunosorbent assay (ELISA) at the Shanghai Xihua Testing Technology Service Co. Ltd. (Shanghai, China). The lower limit of quantification (LLOQ) was 1 ng/mL. The quantitative linear range and the calibration curve assay range were from 100 ng/ml to 3200 ng/ml. The validated largest dilution factor was 6250, so the maximum drug concentration that could be detected was 3200×6250 = 20000000 ng/ml. For the PK analysis, concentrations less than the LLOQ were set to zero. All measurements were accurate to the measure of 97% to 107%. The coefficient of variation for intra- and inter-day precisions was 5.5% to 6.5%.

To determine the PK parameters, a non-compartmental analysis model was used. The concentration-time data comprised the maximum observable serum concentration (C_max_), clearance (CL), half-life (t_1/2_), the volume of distribution (Vd), and AUC from zero to the final quantifiable concentration (AUC_0-t_) and infinity (AUC_0-∞_). The actual sample collection times were considered for the PK analysis. PK parameters were determined using an internally validated software system, Phoenix WinNonLin^®^ v6.4 (Certara L.P., Princeton, NJ, USA).

### Immunogenicity Evaluations

Blood samples were collected at 0.5 h prior to the dosing (pre-dose) and D29, D64, and D134 days post-dose to identify the anti-drug antibodies (ADA) and neutralizing antibodies (NAb). ADA samples were analyzed at the Shanghai Xihua Testing Technology Service Co. Ltd. (Shanghai, China) by conducting two validated, semi-quantitative electrochemiluminescent assays: one each for detecting antibodies against denosumab biosimilar and denosumab. Furthermore, samples with ADA positivity were tested for the presence or absence of neutralizing anti-denosumab biosimilar or anti-denosumab antibodies, by means of validated semi-quantitative electrochemiluminescent NAb assays.

### Pharmacodynamics Evaluations

Blood samples were collected at 0.5 h prior to the dosing (pre-dose) and to D134 days after dosing (**Supplementary Materials**). The serum concentrations of the C-terminal cross-linking telopeptide of type I collagen (CTX1) were evaluated using an enzyme-linked immunosorbent assay (ELISA) at the Shanghai Xihua Testing Technology Service Co. Ltd. (Shanghai, China). The concentration ranged between 0.120 ng/ml and 2.073 ng/ml.

For calculating the PD parameters, a non-compartmental analysis model was utilized. The concentration-time data comprised the minimum observable serum CTX1 concentration (I_min_), the observed time of the lowest serum CTX1 concentration (T_min_), the observed highest percentage of CTX1 inhibition (I_max_), and AUC from zero to the final quantifiable CTX1 concentration (AUEC0–t) and to 134 days (AUEC0–134d). The actual sample collection times were used for the PD analysis. PD parameters were determined through an internally validated software system, Phoenix WinNonLin^®^ v6.4 (Certara L.P., Princeton, NJ, USA).

### Safety Evaluations

Adverse events (AEs) were recorded and graded as per the National Cancer Institute Common Terminology Criteria for Adverse Events V.5.0 (CTCAE 5.0). The AEs were monitored by means of several tests such as physical examination, vital signs, pulse oximetry, electrocardiogram, and common laboratory tests such as urinalysis and chemistry. All AEs were assessed and scored on the basis of their severity and correlation to denosumab and its biosimilars. Subjects with AEs were monitored until the complaint was resolved or stabilized.

### Estimation of Sample Size

As per the current FDA guidelines, the geometric mean ratio (GMR) is set to be 95% to 105% to achieve 90% power (1-β) at the 5% nominal level (α=5%). The coefficient of variation (CV) denotes the inter-subject variability (inter-CV). Because the inter-CV for denosumab is known to be between 32% and 37% (Bekker et al., 2004; Kumagai et al., 2011), initially, the sample size was estimated to be 152, calculated using the PASS Version 11 software (NCSS, Kaysville, UT, USA). Considering the 10% dropout rate, the final sample group size was decided to be 168.

### Statistical Analysis

After the natural logarithmic transformation of C_max_, AUC_0-t_, and AUC_0-∞_, the ANOVA model was used to analyze the difference of the least-square means between denosumab (Xgeva^®^) and its biosimilar. The geometric mean ratio and 90% confidence intervals (CIs) between them were obtained after converting to an inverse natural logarithm. Sex and weight (stratified as ≥66 kg and <66 kg) were included in the ANOVA model as a fixed effect for adjusting for their effect on the bioequivalence. Bioequivalence of PK values between denosumab (Xgeva^®^) and its biosimilar was detected if the 90% CIs for C_max_, AUC_0-t_, and AUC_0-∞_ were recorded to be between 80% and 125%. The PK set population was studied for the PK analysis. This comprised the subjects who were administered a complete dose of the study drug with no deviations in the study protocol. All the subjects who were treated with the study drug were considered for safety analysis. Descriptive statistics were calculated for PK and PD parameters and demographical data. Student’s t-test or Wilcoxon ranks test was used for comparison. All statistical tests were conducted using the SAS 9.1 Statistical Package (SAS Institute Inc., Cary, NC, USA). P<0.05 was considered statistically significant.

## Results

### Subjects

**Figure 1:**
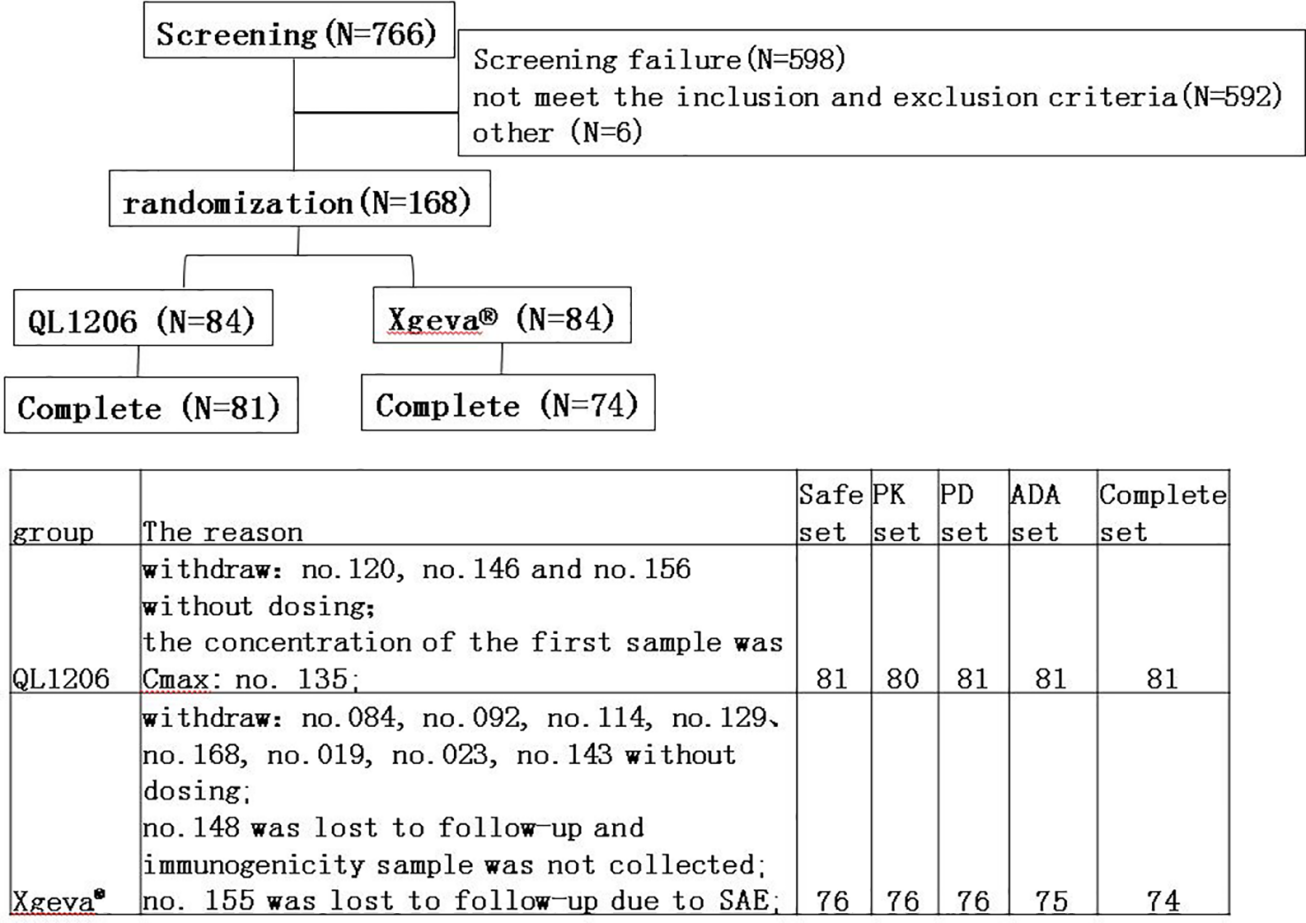
Flow chart of the study.

In total, 766 subjects were screened, of which 168 subjects were registered, and 157 were administered the assigned drugs and were included in the safety analysis set (**Figure 1**). In the QL1206 group, three subjects withdrew their informed consent prior to dosing for personal reasons. Because the concentration of the first sample (1 h post-dosing) was C_max_, subject no. 135 was excluded from the PK analysis set. In the Xgeva^®^ group, eight subjects withdrew their informed consent prior to dosing for personal reasons. One subject from this group was missing at the follow-up, and thus it was not possible to collect that person’s immunogenicity sample. Subject no. 155 was disqualified at follow-up due to a serious adverse event (SAE; a fracture of the left femur, not related to the study drug). The final per-protocol population used in safety, PK, PD, and immunogenicity (ADA) analysis set was comprised of 157, 156, 157, and 156 subjects, respectively (**Figure 1**).

A total of 88 males (56.1%) and 69 females (43.9%) were included in this study, the mean age of whom was 38.2 ± 8.17 years, and the mean BMI 23.7 ± 2.30 kg/m^2^. The demographic and baseline characteristics were comparable between the treatment groups (**Table 1**). Differences between the demographic and baseline parameters between these groups were found insignificant.

**Table 1:** Demographic characteristics of the study.

	QL1206 group (N = 81)	Xgeva^®^ group (N = 76)	total (N = 157)
age(years)	39.5 (8.16)	36.9 (8.02)	38.2 (8.17)
sex			
male	45 (55.6%)	43 (56.6%)	88 (56.1%)
female	36 (44.4%)	33 (43.4%)	69 (43.9%)
ethnic			
HAN	78 (96.3%)	73 (96.1%)	151 (96.2%)
other	3 (3.7%)	3 (3.9%)	6 (3.8%)
BMI (kg/m2)	23.7 (2.44)	23.6 (2.16)	23.7 (2.30)
weight (kg)	23.7 (2.44)	23.6 (2.16)	23.7 (2.30)

### PK Evaluations

**Figure 2:**
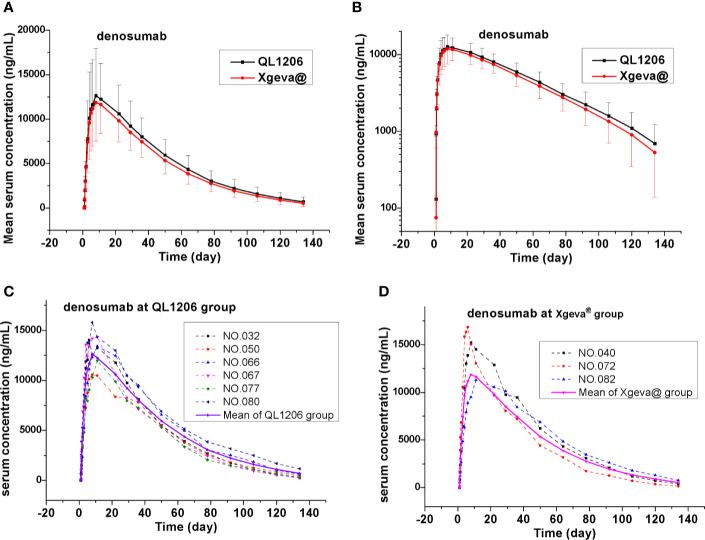
Mean denosumab serum concentration-time profiles in the study. **(A)** Mean values; **(B)** log10 mean values; **(C)** ADA positive subjects (dotted line) and mean values (solid line) at QL1206 group; **(D)** ADA positive subjects (dotted line), and mean values (solid line) of the Xgeva^®^ group.

The PK of denosumab 120 mg in healthy subjects is shown in **Table 2**, **Figure 2**, and **Supplement Figure 1**, which show a similar concentration-time profile and a prolonged absorption phase. The maximum serum concentrations were recorded between 8 and 11 days for the median T_max_. The mean serum concentration-time curve for the denosumab (QL1206 and Xgeva^®^) similarly displayed a gradual reduction in the drug concentration in the serum following the C_max_; thereafter, a quick elimination phase was observed at low concentration (about 1000 ng/mL). The median T_max_ of the QL1206 group was slightly higher than that of the Xgeva^®^ group, although no significant difference (p=0.38) was observed. The mean values of t1/2, C_max_, AUC_0-t_, and AUC_0∞_ of the QL1206 group were slightly greater than those of the Xgeva^®^ group. The mean values of other PK parameters between the QL1206 and Xgeva^®^ groups were observed to be similar (p>0.05).

**Table 2:** Pharmacokinetic parameters of denosumab in each group (mean [CV%] or median [min, max]).

Parameter	QL1206 group	Xgeva^®^ group	p	GMR (90% CI)	GMR (90% CI)^#^	Re-estimated Sample Size
Cmax(ng/mL)	13356.90(39.5%)	12517.89(34.1%)		104.48(94.68–115.29)	104.9(97.12–113.31)	152
*Tmax(hour)	240.00 (72.0, 673.0)	169.00(72.0, 672.0)	0.38			
AUC0-t(hour*ng/mL)	16157045.4(32.1%)	14657546.7(28.8%)		109.35(100.31–119.20)	109.57(101.81–117.92)	180
AUC0-∞(hour*ng/mL)	16815368.3(34.2%)	15306846.5 (29%)		108.08(99.12–117.85)	108.42(100.70–116.73)	180
AUC%Extrap(%)	3.3583(80.1%)	2.9341(97.8%)				
t1/2(hour)	528.24(34.8%)	494.37(34.1%)	0.23			
CL(mL/hour)	8.05(38.4%)	8.55(31.5%)	0.28			
Vd(mL)	5678.8 (30.8%)	5728.3(28.3%)	0.85			

*Tmax: Median (min, max); ^#^adjusting for gender and weight.

Consistent with the mean concentration-time profiles, the mean C_max_, AUC_0-t_, and AUC_0-∞_ approximations and inter-CV values were found comparable in both the QL120 (test) and Xgeva^®^ (reference) groups, with inter-subject CV values ranging from 34.1% to 39.5% for C_max_, 28.8% to 32.1% for AUC_0-t_, and 29% to 34.2% for AUC_0-∞_ (**Table 2**). The 90% CIs were determined by using “variable analysis method” (ANOVA), “pair” experiments with two parallel groups of treatments through the SAS computer program. The results are presented in **Table 2**. The 90% CI values of the test-to-reference ratios for C_max_, AUC_0-t_, and AUC_0-∞_ were recorded to be within the bioequivalence window range of 80%–125% when compared to that of Xgeva^®^. The inter-CV of exposure ranged from 28.8% to 39.5%. The 90% CI value was found to be wider when the inter-CV value was larger. The two groups were still bioequivalent even after adjusting for the values of gender and weight of the subjects. The sample size of the study was re-estimated based on their bioequivalence analysis results (geometric mean ratio [GMR] and inter-CV values), which was recorded to be 152 to 182, similar to the size at the time of enrollment in this study (**Table 2**). In both the QL1206 and Xgeva^®^ groups, the concentration of the denosumab-time profile in male subjects was higher than that in female subjects (**Supplement Figure 2**).

### Pharmacodynamics Evaluations

**Figure 3:**
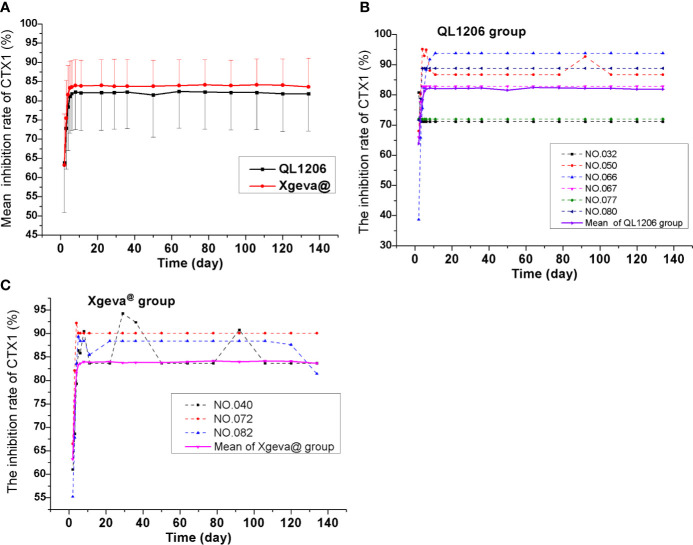
Mean serum inhibiting rates of CTX1–time profiles in the study. **(A)** Mean values; **(B)** ADA positive subjects (dotted line) and mean values (solid line) of the QL1206 group; **(C)** ADA positive subjects (dotted line) and mean values (solid line) of the Xgeva^®^ group.

The pharmacodynamics of QL1206 and Xgeva^®^ drugs were examined. A single dose (120 mg) of the study drug subcutaneously lead to a rapid and profound (up to about 80% at 48 h) and sustained (up to 134 days) decline in the CTX1 concentration in serum. At day 134, a mean change from the baseline of 81.84% was recorded to 83.64% in both the QL1206 and Xgeva^®^ groups. The median T_min_ values of CTX1 of the two groups was the same, in that both were 48 h. The maximum inhibition percentage of CTX1 in both groups was about 80%, and the difference in other pharmacodynamic parameters between the two groups was estimated to be within 5%, as exhibited in **Table 3** and **Figure 3**. In both the QL1206 and Xgeva^®^ groups, the pharmacodynamics index (CTX1 inhibition rate)-time profile was also higher in male subjects than that in female subjects (**Supplement Figure 3**).

**Table 3:** Pharmacodynamic parameters of CTX1 in each group (mean [CV%] or median [min, max]).

parameter	QL1206 group	Xgeva^®^ group
I_min_(ng/mL)	0.0622(22.4%)	0.0613(18.2%)
*T_min_(hour)	48(24.0,1512.0)	48(24.0,2520.8)
I_max_(%)	82.45(11.6%)	84.22(7.8%)
AUEC_0-t_(hour*%)	260293.4(11.6%)	262167.4(13.3%)
AUEC_0-134d_(hour*%)	260219.7(11.6%)	266283.2(7.9%)

*Tmin: Median (min, max).

### Immunogenicity Evaluations

ADA was recorded to be negative among all subjects prior to dosing. Subjects to the count of 7.4% (6/81) and 4.0% (3/76) of the denosumab biosimilar and reference groups were, respectively, found to be positive for ADA at a certain time during the study period, who turned out to be negative at the last follow-up. Nevertheless, none of them were found positive for NAb. Denosumab biosimilar had a similar ADA profile to that of Xgeva^®^ in this study. The serum concentration-time curve of denosumab and its biosimilar of ADA positive subjects were similar to the mean serum concentration-time curve of each group (**Figures 2C, D**). The CTX1 inhibition rate-time profiles of ADA positive subjects were also similar to the mean CTX1 inhibition rate-time curve of each group. These findings indicated that the ADA did not influence the PK and PD of denosumab (**Figures 3B, C**).

### Safety Evaluations

The AEs among 75 subjects (255 times, 92.6% [75/81]) of the QL1206 group and 71 subjects (241 times, 93.4% [71/76]) of the Xgeva^®^ group were recorded. The incidence was the same in the two groups. No deaths or discontinuations were due to AEs except a case of left femur fracture (subject no. 155, SAE) occurred. Most AEs were grade 1 or 2, and no abnormal reactions at the injection site were observed, except among three subjects of the QL1206 group. A red rash was observed at the injection site at 12 h to 48 h post-dosing (probably related to the drug), from which they recovered spontaneously. AE (greater than grade 3 of CTCAE 5.0 criterion) was observed to occur among nine subjects (16 times, with an incidence rate of 11.1%) of the QL1206 group and five (7 times, with an incidence rate of 6.6%) of the Xgeva^®^ group related to the study drug, which is mainly a decline in the blood phosphorus level. The rest of the cases included hyperuricemia, hypertension, and periodontitis, all of which were infrequent. The AEs with an incidence of higher than 5% linked to the study drug were mainly a decline in blood calcium and blood phosphorus level, hypertriglyceridemia, and so on. The most frequently occurring AE was a decline in blood calcium level, with an incidence of 72.8% as against 72.4% in the QL1206 and Xgeva^®^ groups, respectively (**Supplementary Table 1**).

Two SAEs, namely, spontaneous abortion (subject no. 077), assumed to be likely linked to the study drug, and left femur fracture (subject no. 155), caused by a traffic accident, declared to be definitely not related to the study drug, exited the study, and the other AEs were followed up until they recovered or stabilized. Most AEs recovered automatically without any treatment, and about 31 (19.7%) subjects used concomitant drugs due to AEs in the study, such as calcium tablets, antibacterial drugs, and analgesics, which were similar in the two groups.

## Discussion

Denosumab appeared to be well-tolerated, and no significant safety issues were observed. Denosumab (QL1206 and Xgeva^®^) is a potent antiresorptive agent, and hypocalcemia was not significant. Xgeva^®^ drug label lists the most common AEs to be hypocalcemia, back pain, hypercholesterolemia, musculoskeletal pain, cystitis, arthralgia, and nasopharyngitis, which agreed with the observations of this study. Denosumab is known to bind to RANKL, inhibiting the formation, function, and survival of osteoclasts; reducing bone resorption; and increasing bone mass, which leads to a smaller amount of calcium released from the bones, eventually causing hypocalcemia, followed by affecting the parathyroid gland, thus contributing to reduced phosphorus (Bekker et al., 2004; Evenepoel et al., 2014). Earlier studies have reported 83% (19/23) of subjects administered Xgeva^®^ to have experienced drug-induced AEs (Chen et al., 2018). As these AEs were nonspecific and occurred equally in both the biosimilar and Xgeva^®^ groups, it appears that denosumab and its biosimilar drug have comparable tolerability profiles among healthy subjects (Li et al., 2015).

Bekker investigated the PK characters of AMG162 (denosumab) of the dosage ranging from 0.01 to 3 mg/kg among postmenopausal women. A prolonged elimination phase, characterized by t1/2 that increased with the dose to a maximum of 32 days, and a more rapid terminal elimination phase observed at concentrations of <1,000 ng/ml with a t1/2 that increased from 5 to 10 days with an increasing dose from 0.01 to 3.0 mg/kg, demonstrating the nonlinear PK characters (Bekker et al., 2004). In this study, the denosumab concentration curve (log unit), at high concentrations, drops like a straight line, and the elimination of denosumab is quicker, and the curve drops steeper, at low concentrations (about 1,000 ng/ml), which is in accordance with the above nonlinear PK characters of denosumab.

In certain earlier reports on Caucasians (Evenepoel et al., 2014; AmgenXgeva, 2020), provided in **Supplementary Table 2**, the observed median T_max_ was 6.9 to 10 days (7–10 days in this study), mean C_max_ was 11.8–14.1 μg/mL (12.5–13.36 μg/mL in this study), and mean t1/2 was 22.4 to 25.8 days (20.6–22 days in this study) on treatment with denosumab 120 mg. The C_max_ and Vd values were also alike among both the Chinese and Caucasians. CL value was recorded slightly higher among Chinese (333.3–356.3 mL/day) than that among Caucasians (147.5 mL/day), and AUC_0-t_ value was observed to be slightly lower among the Chinese (610.7–673.2 day*μg/mL) than that among Caucasians (775.4 day*μg/mL). Nevertheless, very slight differences, especially in the AUC and the different detection methods, may have been the cause. Largely, the PK results of this study were comparable with those of the earlier reports, indicating no clinically relevant differences between the PK profiles of Chinese and Caucasians (Cai et al., 2012; Liu et al., 2015).

CTX1 acts as a specific marker of bone reabsorption, the serum levels of which are proportional and suggestive of osteoclastic activity at the time of blood sampling. In earlier clinical studies, a treatment dosage of 60 mg denosumab leads to a reduction in the bone resorption marker serum type 1 C-telopeptide (CTX1) by approximately 85% in 3 days, with maximal reductions occurring in a month’s time among Caucasians (Amgen, 2019), which agreed with the findings of this study (a rapid and profound decrease in CTX1of up to 80% at 48 h). CTX1 levels were below the assay quantitation limit (0.049 ng/mL) in 39% to 68% of patients from 1 to 3 months post-dosing with 60 mg denosumab. At the end of each dosing interval (administer 60 mg every 6 months, about 180 days), CTX1 reductions were partially attenuated from a maximal reduction of ≥87% to ≥45% (range: 45–80%), as serum levels of denosumab reduced, indicating the reversibility of the effects of 60 mg denosumab on bone remodeling (Evenepoel et al., 2014; Amgen, 2019). In this study, the dosage was set at 120 mg, and the subjects were followed up to 134 days. Thus, the inhibition rate of CTX1 remained at 80%, and recovery was not observed, due to the higher dosage and shorter follow-up time in this study. The concentration of monoclonal antibody diminished significantly in the latter phase; however, CTX1 did not recover. Then the PK and PD models also could not be established. CTX1 is the degradation product of type I collagen during bone resorption. When the concentration of denosumab in the blood is very low or returns to zero, the link of RANKL between osteoblasts and osteoclasts could not be quickly restored, and osteoblasts cannot be immediately absorbed. Therefore, the concentration of CTX1 is still very low, and the inhibition rate of CTX1 is still high at the end of follow-up in this study, which indirectly proves the persistent efficacy of denosumab (Amgen, 2019). Three inhibition rate values after dosing (2–4 days) showed that the efficacy increased rapidly and almost reached the platform of the maximum inhibition rate in 5–8 days after dosing (**Figure 3**).

In both the QL1206 and Xgeva^®^ groups, the concentration of denosumab-time profiles in male subjects was higher than that in female subjects. Meanwhile, the pharmacodynamics index (CTX1 inhibition rate) was also higher in male subjects than that in female subjects. Still, the two above indexes between the QL1206 and Xgeva^®^ groups were very close to each other in the same sex, which was similar to the results of previous population pharmacokinetic meta-analysis of denosumab in healthy subjects and postmenopausal women (Sutjandra et al., 2011). The mean RANKL level is 3.97–5.8 nmol/L in healthy young women and pre- and postmenopausal Chinese women; it is 1.69 nmol/L in osteoarthritic males. Assuming stoichiometry of 1:1 binding for denosumab and RANKL, the rest denosumab concentrations will be higher in the male than the female subjects at the same dosage. Then CTX1 inhibition rate was also higher in males (Sutjandra et al., 2011). This indicated that the ratio of male to female between the reference drug and the test drug should be similar in the clinical trial.

Overall, 31 subjects (19.7%) were treated for various AEs, such as with calcium tablets, in this study. The concomitant medication used for the two groups was similar. As denosumab is a remodeling monoclonal antibody, its metabolic pathway is usually the swallowing function of mesenchymal cells, rather than *via* the liver’s metabolic activity, such as Cytochrome P450 (Chen et al., 2018). Thus, the above concomitant medication hardly influenced the PK characters of denosumab.

In this study, immunogenicity was analyzed, along with the efficacy and safety of the drugs. The overall ADA positive rate was found to be slightly higher in this study than that of the earlier studies, and the rate is similar in both denosumab biosimilar and reference drug groups. The ADA rates have been recorded to be 7.4% (6/81) as against 4.0% (3/76) versus 0.5% (1/199) in the QL1206 and Xgeva^®^ groups of this study, and Xgeva^®^ in multiple myeloma patients (Amgen, 2017; AmgenXgeva, 2020). The observed incidence of antibody (including neutralizing antibody) positivity in an assay may be affected by several factors such as assay methodology, sample handling, the timing of sample collection, concomitant medications, and underlying disease. Owing to such reasons, comparison of the incidence of antibodies to denosumab in various studies may be misleading. Neutralizing antibodies were not produced in any subject. The production of ADA had neither any significant effect on PK parameters such as serum concentration (**Figures 2C, D**) nor on therapeutic effect (**Figures 3B, C**). In this study, no indication of clear clinical significance for the positive detection of ADA was found. Most significantly, denosumab PK and PD were not observed to be influenced by the binding antibodies formation, and denosumab demonstrates low immunogenicity in humans [FDA(CHINA), 2016].

This phase I study confirmed that denosumab biosimilar (QL1206) has similar PK and PD profiles to those of Xgeva^®^ examined in healthy subjects. The 90% CI values of the test-to-reference ratios for C_max_ and AUC were observed to be within the predefined bioequivalence approved range of 80% to 125% for the biosimilar in comparison with Xgeva^®^. The PK and PD similarities between the licensed Xgeva^®^ products and those of the biosimilar substantiate the use of the biosimilars in phase III clinical studies (Davit et al., 2008; Karalis et al., 2012). The inter-CV value of denosumab among Chinese subjects is high, and it is recommended that in future studies, the sample size of 152 to 180 subjects is sufficient to study the bioequivalence of denosumab biosimilar in each group, considering inter-CV (28.8–39.5%) (Liu et al., 2012; Al-Sabbagh et al., 2016; Hammami et al., 2017).

## Conclusion

This study evidenced that the PK and PD profiles of the denosumab biosimilar (QL1206) were the same as those of Xgeva^®^. The denosumab biosimilar exhibited a similar ADA profile with no detection of Nab and a comparable safety profile in comparison with the reference drug. The inter-CV of denosumab was found to be higher. These data support the clinical application of QL1206 as a denosumab biosimilar.

## Data Availability Statement

The raw data supporting the conclusions of this article will be made available by the authors, without undue reservation, to any qualified researcher. Requests to access these datasets should be directed to yanhuad2019@163.com.

## Ethics Statement

The Institutional Review Board of the First Hospital of Jilin University reviewed and approved the final protocol, any amendments, and informed consent documentation. The study agreed with guidelines of the Declaration of Helsinki, International Conference on Harmonization Good Clinical Practice Guidelines, and local regulatory requirements. Written informed consent was obtained from all subjects prior to including them in the study.

## Author Contributions

HZ, MW, XZ, and YD designed the experiment. CYL, XL, JS, CJL, QL, WW and JN performed the clinic trials. HZ, QL, WW and JN analyzed the data. HZ and YD wrote and edited the paper and drew the figures. All authors contributed to the article and approved the submitted version.

## Funding

This work was supported by the National Major Scientific and Technological Special Project for Significant New Drug Development during the Thirteenth Five-Year Plan Period of China (Project: 2017ZX09304004, 2017ZX09101001-002-004), the National Natural Science Foundation of China (Project: 81602897), and the National Major Scientific and Technological Special Project for “Significant New Drugs Development” during the Thirteenth Five-Year Plan Period of China (No. 2018ZX09301007005). The authors declare that this study received funding from the Qilu Pharmaceutical Co., Ltd, China. The funder was not involved in the study design, collection, analysis, interpretation of data, the writing of this article or the decision to submit it for publication.

## Conflict of Interest

The authors declare that the research was conducted in the absence of any commercial or financial relationships that could be construed as a potential conflict of interest.
