# Supplementary material for: A Phase I, Randomized, Single-Dose Study to Evaluate the Biosimilarity of QL1206 to Denosumab Among Chinese Healthy Subjects
Source: Front Pharmacol. 2020 Oct 8;11:01329. doi: 10.3389/fphar.2020.01329 (PMC7580200; doi:10.3389/fphar.2020.01329)
Supplement: Supplementary file 1 [file DataSheet_1.docx]

**Supplement**

**1. Methods**

**1.1 Supplement inclusion criteria**:

Subjects had not used any drugs influencing bone metabolism, such as bisphosphonates and parathyroid hormones, during the 12 months before the commencement of the study. Females were neither pregnant nor lactating or using oral contraceptives and had no pregnancy planned for the 6 months post the study period. All subjects were instructed to use condoms during the study period.

**1.2 Supplement Exclusion criteria**:

Subjects with evidence or history of clinically significant diseases, history of cancer, hypertension, or heart disease (defined in terms of QTc>450ms); subjects who had received denosumab treatment earlier or were positive for denosumab antibody. Individuals with serum calcium levels exceeding the normal values during the screening period or subjects who used calcium supplements were also not included.

**1.3 The collection time points of different indicators**

Blood samples for PK evaluation were collected at 0.5 h prior to dosing (pre-dose), and 1h, 4h, 8h, 12h, 24h (D2), 48h (D3), D4, D5, D6, D8, D11, D22, D29, D36, D50, D64, D78, D92, D106, D120, and D134 days postdosing. The blood samples were stored at room temperature for 30 min to clot and then centrifuged at 1500-1700g for about 15 min at 2–8°C. The serum was stored at −70°C for further analysis.

Blood samples for PD evaluation were collected at 0.5 h prior to the dosing (pre-dose), and D2, D3, D4, D5, D6, D8, D11, D22, D29, D36, D50, D64, D78, D92, D106, D120, and D134 days after dosing. The biological sample collection method is similar to that of the PK samples.

**Supplementary Table 1. Treatment-emergent adverse drug reaction (number of events, the number [%] of subjects, more than 5%)**

|  | QL1206 group (N = 81) | | Xgeva^® group (N = 76)^ | |
| --- | --- | --- | --- | --- |
|  | n (%) | [nAE] | n (%) | [nAE] |
| **total** | 75 (92.6%) | 255 | 71 (93.4%) | 241 |
| **Metabolic and nutritional diseases** | 65 (80.2%) | 139 | 59 (77.6%) | 104 |
| decline in blood calcium level | 59 (72.8%) | 113 | 55 (72.4%) | 85 |
| hypertriglyceridemia | 7 (8.6%) | 9 | 7 (9.2%) | 8 |
| decline in blood phosphorus level | 5 (6.2%) | 9 | 5 (6.6%) | 7 |
| **Various musculoskeletal and connective tissue abnormalities** | 26 (32.1%) | 37 | 23 (30.3%) | 31 |
| Muscle pain | 9 (11.1%) | 10 | 4 (5.3%) | 4 |
| Muscle cramps | 7 (8.6%) | 12 | 8 (10.5%) | 11 |
| Joint pain | 6 (7.4%) | 6 | 1 (1.3%) | 1 |
| Back pain | 5 (6.2%) | 5 | 7 (9.2%) | 8 |
| Body unwell | 0 | 0 | 5 (6.6%) | 5 |
| **Abnormal value detected by laboratory** | 18 (22.2%) | 32 | 28 (36.8%) | 55 |
| Alanine aminotransferase elevated | 5 (6.2%) | 7 | 7 (9.2%) | 8 |
| Aspartate aminotransferase elevated | 3 (3.7%) | 4 | 5 (6.6%) | 6 |
| white blood cell count elevated | 1 (1.2%) | 1 | 7 (9.2%) | 7 |
| neutrophil count elevated | 1 (1.2%) | 1 | 6 (7.9%) | 6 |
| Bilirubin elevated | 0 | 0 | 4 (5.3%) | 6 |
| **Respiratory, chest and mediastinal diseases** | 10 (12.3%) | 13 | 20 (26.3%) | 22 |
| cough | 5 (6.2%) | 6 | 13 (17.1%) | 13 |
| running nose | 2 (2.5%) | 3 | 5 (6.6%) | 5 |
| **Gastrointestinal disease** | 9 (11.1%) | 11 | 12 (15.8%) | 13 |
| toothache | 7 (8.6%) | 8 | 10 (13.2%) | 11 |

**Supplementary Table 2.Comparison of serum pharmacokinetic parameters and literature (mean [CV%] or median[min, max])**

| Parameter | PK data in this study | | Amgen PK data[4] | Comparison of CP4 and CP2 batches from amgen[16] | |
| --- | --- | --- | --- | --- | --- |
|  | QL1206 120mg(n=80) | Xgeva^®^ 120mg(n=76) | 120mg(n=23) | CP4 120mg(n=71) | CP2 120mg(n=71) |
| C_max(μg/mL)_ | 13.36(39.5) | 12.5(34.1) | 14.1(22.0) | 12.7(27.8) | 11.8(29.7) |
| AUC_(0-t) day*μg/Ml_ | 673.2(32.1) | 610.7(28.8) | 775.4(23.2) | / | / |
| AUC_(0-∞) day*μg/mL_ | 700.6(34.2) | 637.8(29.0) | 813.5(25.0) | / | / |
| AUC_(0-18week) day*μg/mL_ | / | / | / | 579(32.8) | 555(29.9) |
| AUC_(0-16week)day*μg/mL_ | / | / | 752.3(22.7) | / | / |
| T_max_(day) | 10(3.0-28.0) | 7(3.0-28.0) | 10.0(4.0-27.0) | 6.9(2-14) | 7.0(3.0-2.1) |
| T_1/2_(day) | 22(34.8) | 20.6(34.1) | 25.8(29.1) | 23.9(42.2) | 22.4(45.5) |
| CL(mL/day) | 333.3(38.4) | 356.3(31.5) | 147.5(25.0) | / | / |
| Vd(mL) | 5678.8(30.8) | 5728.3(28.3) | 5495.9(25.4) | / | / |


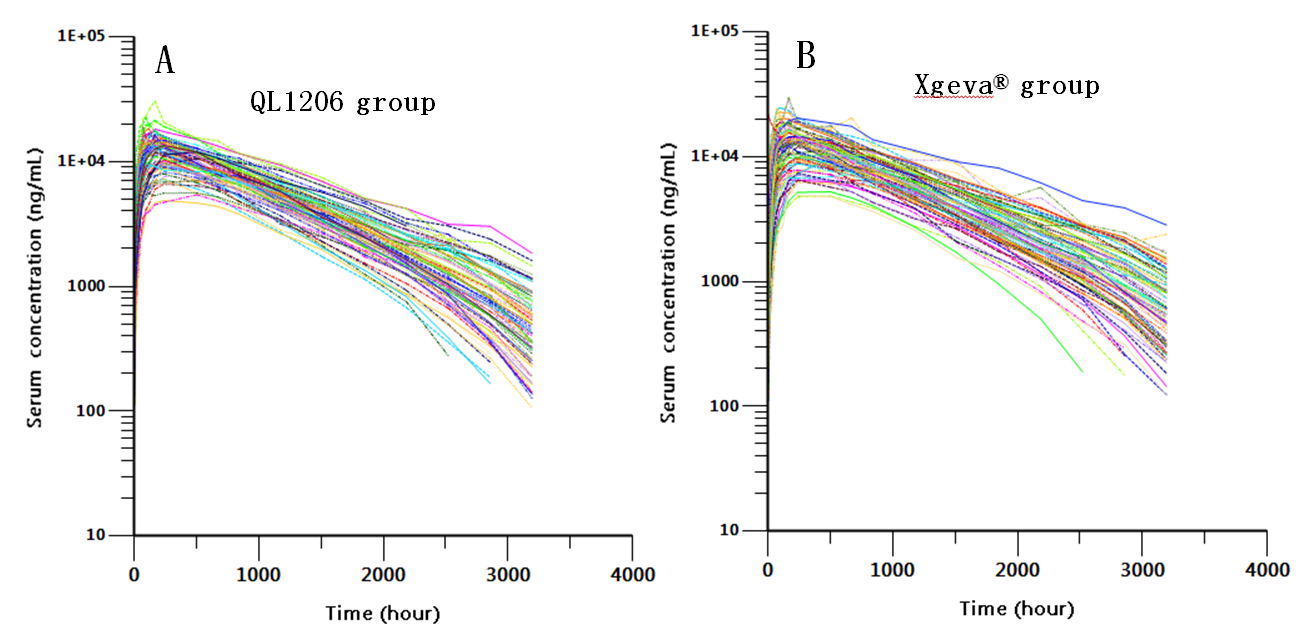


**Supplement Figure.1 Individual serum concentration of denosumab biosimilar or Xgeva®; each line represents a subject**


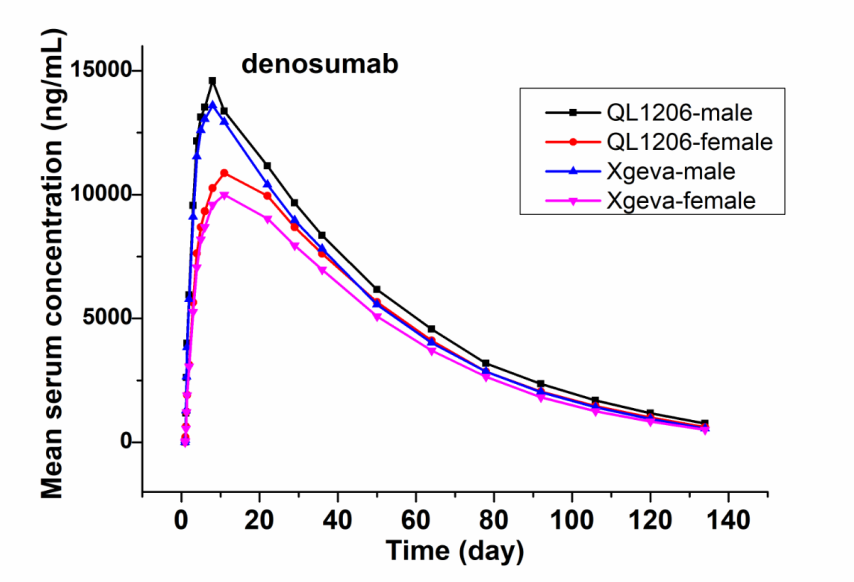


**Supplement Figure.2 Mean denosumab serum concentration-time profiles with sex stratification in the study.**


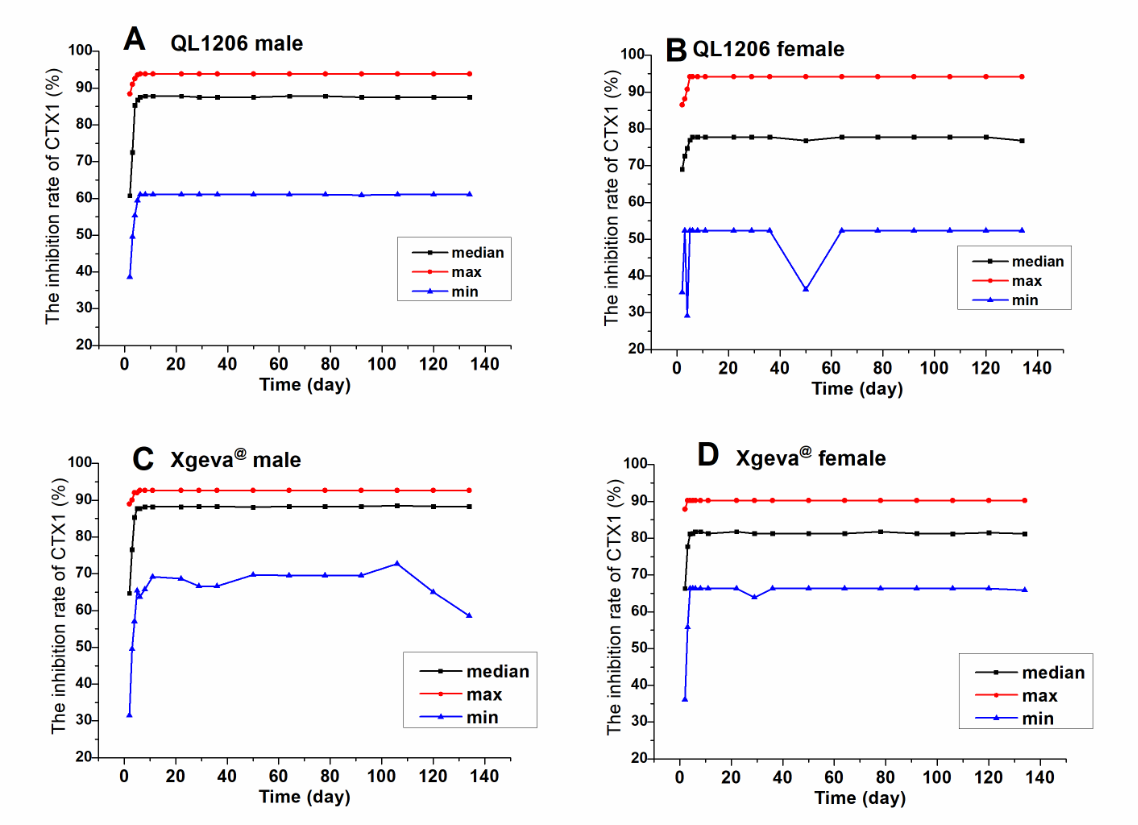


**Supplement Figure.3 Median serum inhibiting rate of CTX1-time profiles with sex stratification in the study.**
